# Alternative Approach for Cerebral Protection during Complex Aortic Arch and Redo Surgery

**DOI:** 10.3390/jcdd8080086

**Published:** 2021-07-27

**Authors:** Massimo Capoccia, Christoph A. Nienaber, Maziar Mireskandari, Michael Sabetai, Christopher Young, Nicholas J. Cheshire, Ulrich P. Rosendahl

**Affiliations:** 1 Aortic Centre, Royal Brompton Hospital, London SW3 6NP, UK; C.Nienaber@rbht.nhs.uk (C.A.N.); M.Mireskandari@rbht.nhs.uk (M.M.); N.Cheshire@rbht.nhs.uk (N.J.C.); U.Rosendahl@rbht.nhs.uk (U.P.R.); 2 Cardiac Surgery, Guy’s & St. Thomas’ Hospital, London SE1 9RS, UK; Michael.Sabetai@gstt.nhs.uk (M.S.); chris@heartfix.net (C.Y.)

**Keywords:** arch surgery, cerebral protection, redo surgery

## Abstract

Total arch replacement remains a very demanding surgical procedure. It can be associated with reasonable long-term outcomes but carries serious perioperative complications. Aortic arch surgery has progressed in recent years to a wider adoption of reproducible and reliable techniques. Conventional open, surgical aortic arch replacement is currently offered to the majority of patients, although hybrid and wholly endovascular techniques are gaining popularity. With regards to open arch replacement, the nuances of surgical technique, the mode of cannulation and the optimal cerebral protection protocols remain a matter of debate. We propose an alternative cannulation approach facilitated by the cooperation between cardiac and vascular surgeons. A three-way arterial cannulation including both carotid arteries and the femoral artery (or ascending aorta) is the key feature of this approach. A case series of complex patients is presented to show both the feasibility and relative safety of a standardised new approach with a 100% technical success rate and a 16% 30-day mortality. The three-way cannulation approach may have a role to play for complex and extensive procedures requiring prolonged cerebral protection. We believe that a shared skill set from cardiac and vascular specialists is essential for the safe management and successful outcomes using this adaptive technique.

## 1. Introduction

Total arch replacement surgery continues to be a challenge. Durability after a successful total arch repair has been shown to be acceptable, but it still carries the risk of potentially devastating perioperative complications. Since the early attempts [1,2], aortic arch surgery has progressed with multiple technical adaptations. Classic conventional aortic arch replacement is currently being offered to the majority of patients, but hybrid and endovascular techniques have gained popularity. These less invasive approaches can often be as technically challenging as open surgery with stroke and endo-leaks as early limiting factors and less favourable mid- to long-term results [3]. At the present time, reported mid-term outcomes and intra-operative complication rates with both hybrid and conventional aortic arch surgery remain heterogeneous and depend on centre experience and patient suitability [4].

Median sternotomy remains the most prevalent access for classic surgical aortic arch replacement. When central aortic cannulation is not feasible, arterial cannulation through one of the femoral arteries (either directly or via an end-to-side Dacron graft) is appropriate. Axillary artery cannulation [5] is gaining interest, but it is not without other risks and complications [3,6]. The innominate [7] and the common carotid artery [8] have also been considered as cannulation sites.

Open repair with the elephant trunk technique [9] under hypothermic circulatory arrest is the most frequently reported technique with reasonable long-term results [10,11]. More recently, the frozen elephant trunk using the Thoraflex^TM^ hybrid device (Vascutek, Terumo) has become a popular choice, which allows subsequent endovascular procedures [12,13]. Hybrid repair encompasses various techniques to debranch the aortic arch, thereby creating an appropriate landing zone for an additional or staged endovascular intervention.

In addition, cerebral protection is a constant matter of debate. Hypothermia significantly reduces oxygen demand and cerebral metabolism. Therefore, profound hypothermia (18 °C) with total circulatory arrest as the only mode of protection became widely accepted [14] at the expense of some neurological injury as the metabolism was never reduced to zero [15]. The initial enthusiasm for the use of retrograde cerebral perfusion as an additional method of protection [16] has been challenged by its unpredictable effects and ability to provide adequate cerebral capillary perfusion [17,18,19,20,21]. Although retrograde cerebral perfusion is still used by some groups [22,23], antegrade selective cerebral perfusion has gained more acceptance [24,25,26]. The combination of antegrade selective cerebral perfusion with moderate hypothermia [24,27] has reduced the potential for neurological injury by allowing more time for repair [28,29,30]. Nevertheless, controversy remains whether unilateral or bilateral perfusion should be considered [31,32,33] with particular reference to the completeness of the circle of Willis [34]. More recently, the “branch-first” technique without circulatory arrest or deep hypothermia has been proposed [35,36].

Given the above conflicting considerations, we propose an alternative approach facilitated by the use of the combined skill set of cardiac and vascular specialists. The carotid-to-carotid cannulation through an end-to-side Dacron graft combined with a femoral or central cannulation provides a three-way arterial access offering safe perfusion to both cerebral hemispheres and enhanced flexibility with regard to hypothermia and the use of circulatory arrest.

## 2. Patients and Methods

The following case series represents examples of complex procedures in which we have applied the three-way cannulation approach. Here we have mainly addressed the technical aspects of our access strategy for aortic arch replacement and redo surgery.

The case selection and discussion take place at our Aortic MDT and are based on our standard approach to treatment, regardless of the case complexity.

Arterial and venous monitoring is usually obtained through the left radial artery and the right internal jugular vein. The chest is routinely entered through a median sternotomy. Trans-oesophageal echocardiographic monitoring is advised in view of its additional information. The cardiopulmonary bypass circuit consists of a three-way arterial cannulation including both carotid arteries and the femoral artery (Figure 4). Central cannulation through the ascending aorta is also an option when feasible. A 10 mm Dacron graft is sutured to each carotid artery to facilitate cannulation and allow the transposition into the chest later with anastomosis to the ascending aorta (Figure 5). We prefer a 10 mm Dacron graft to the femoral artery when required. Venous drainage is addressed with a multi-stage cannula through the femoral vein and additional tubing connection should further drainage be required through the superior vena cava. If central cannulation is considered, then venous drainage would be achieved with a single, two-stage cannula through the right atrium. Cerebral blood flow is delivered at 10 mL/kg/min with a perfusion pressure between 50 and 70 mmHg, as previously described by Kazui et al. [24]. A clamp on the innominate artery maintains blood flow in the right vertebral artery for further brain protection. The Trendelenburg position and CO_2_ flow are used to reduce the risk of an air embolism. The setting of the circuit with 3/8 tubing connectors directly inserted into the 10 mm Dacron grafts is designed for total control by the perfusionist with the ability to measure blood flow and redirect perfusion when required. This is particularly important when cerebral blood flow needs re-arrangement to avoid excessive flow and pressure. Continuous, non-invasive monitoring of the cerebral oxygen saturation (INVOS system) and somatosensory evoked potentials are recommended. We have mainly used the Thoraflex^TM^ hybrid device (Vascutek, Terumo, Inchinnan, Glasgow, UK) for arch reconstruction based on the frozen elephant trunk technique. An alternative device is the E-vita OPEN PLUS (JOTEC, Hechingen, Germany). The Siena collared graft (Vascutek, Terumo) has been used when a conventional elephant trunk technique was considered.

## 3. Results

Table 1 gives the clinical and operative data with an overview of the postoperative outcome of 12 patients who underwent complex repair using the proposed approach.

**Figure 1:**
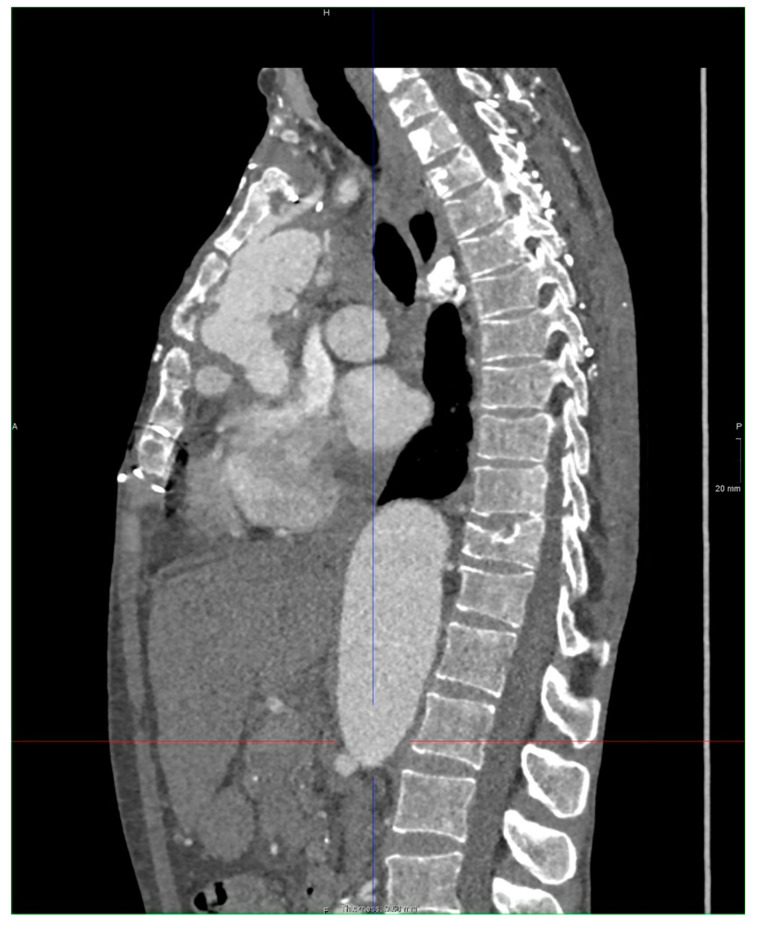
Patient 8. Infected Dacron graft in the ascending aortic position with contained rupture and pseudo-aneurysm formation adherent to the sternum.

**Figure 2:**
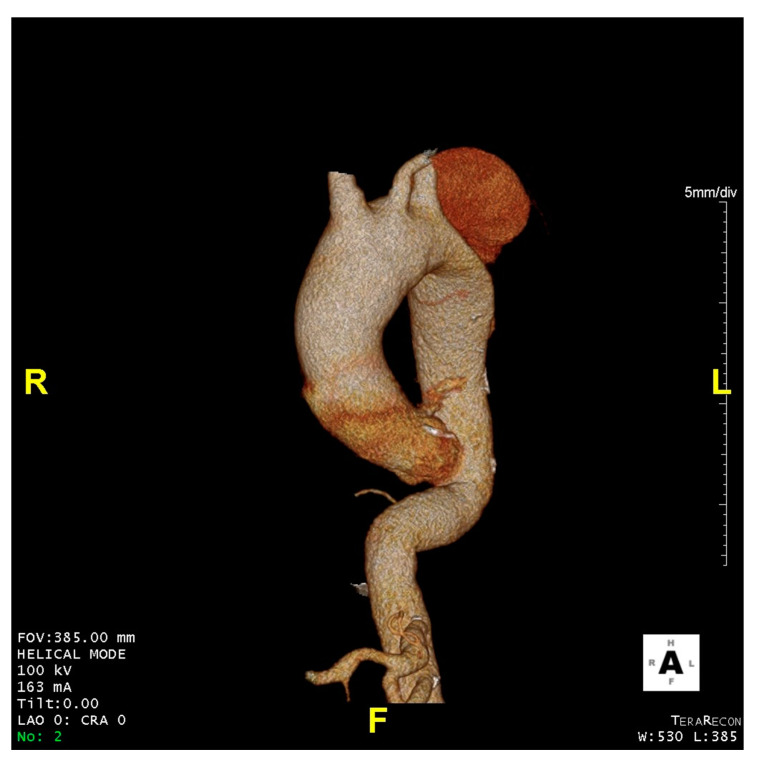
Patient 6. Aneurysm of the ascending aorta and distal aortic arch (8 cm) in previous acute type B aortic dissection treated conservatively.

**Figure 3:**
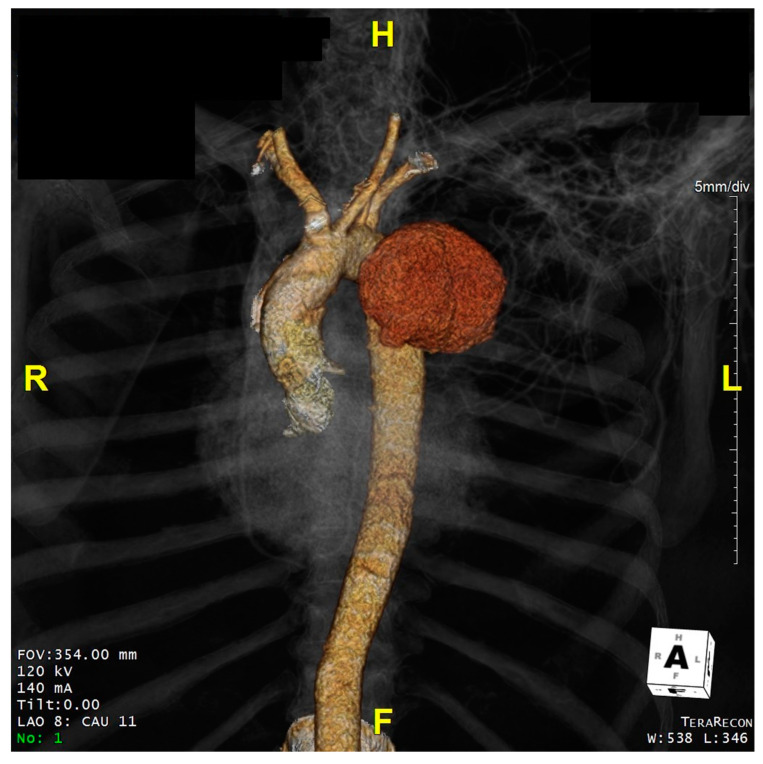
Patient 7. Severe aneurysmatic dilatation of the distal aortic arch and proximal descending thoracic aorta with compression of the left branch of the pulmonary artery.

**Figure 4:**
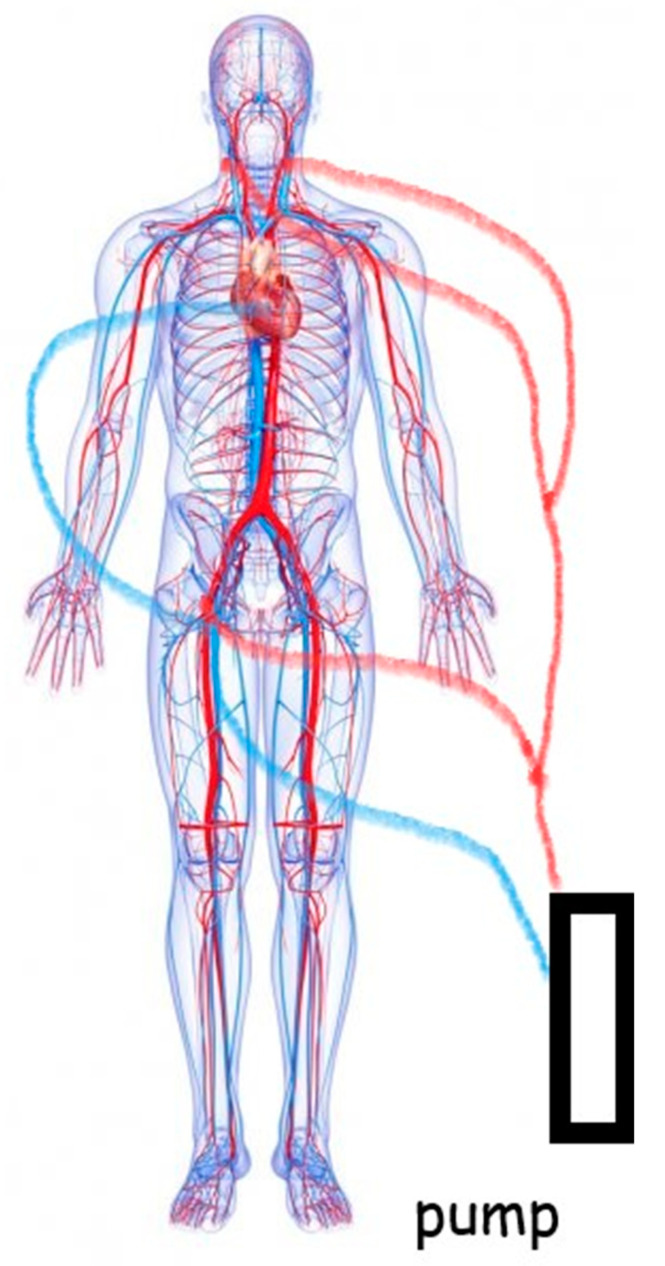
Completion of the cardiopulmonary bypass circuit with the three-way arterial cannulation approach. The red lines represent the arterial configuration. The blue lines show the venous configuration with an extension should cannulation of the superior vena cava be required for further drainage.

**Figure 5:**
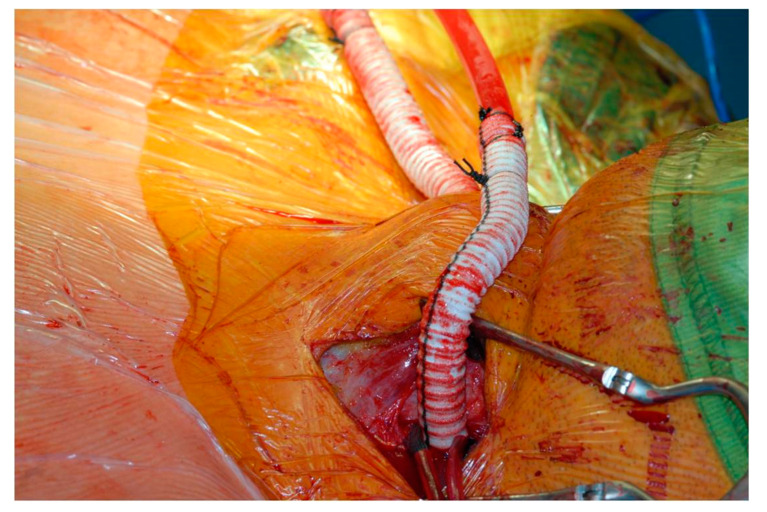
Completion of the end-to-side anastomosis for cerebral perfusion.

Figure 1, Figure 2 and Figure 3 show key preoperative images of some patients. Figure 4 gives a schematic representation of the circuit with the three-way arterial cannulation. Figure 5 is a key intra-operative detail.

## 4. Discussion

It is well known that the management of aortic arch disease either in the context of acute type A aortic dissection or in the presence of aneurysmatic disease is a challenging task and a subject of conflicting debate [37,38]. Open repair for aortic arch disease is still considered the standard of care in high volume centres, although the advent of endovascular treatment has led to a reset in the “modus operandi”, particularly when a lack of fitness and comorbidities may preclude a traditional surgical approach [39]. For such compelling patients, the role of the aortic team becomes even more important by combining the skill set of various specialists [40,41].

Stroke remains the most important complication in open, hybrid and total endovascular approaches. For open surgery, the increased use of selective antegrade cerebral perfusion (SACP) with warmer circulatory arrest (24–28 °C) has contributed significantly to improved stroke and lower death rates, although there is no clear consensus about the optimal protection strategy.

The alternative cannulation approach described in this context may have advantages for highly complex cases of open aortic arch surgery, particularly for complex procedures in patients with difficult access and significant comorbidities. The technique is also applicable to redo cases where the aorta is completely attached to the inner plate of the sternum with a high likelihood of injury during resternotomy. Patient 1, patient 5 and patient 8 in our series are three typical examples. The use of 10 mm Dacron grafts to the carotid arteries allows great flexibility with tunnelling under the clavicle and anastomoses to the proximal arch graft to reconstruct the head vessels; this may make the mediastinal operation easier and give more surgical options in the chest. We prefer a 10 mm size graft in view of its better fit to the perfusion cannula and the increased surface area. Potential kinking can be avoided by keeping the grafts loose with a lazy “S” course during the transposition into the chest. The limited oblique or transversal incisions at the base of the neck are cosmetically acceptable following patients’ feedback.

The fashioning of tubular grafts to both carotid arteries allows cerebral perfusion to be maintained all of the time in a secure manner and without the need of additional cannulation through the supra-aortic vessels following incision and resection of the aortic arch. We believe that bilateral carotid cannulation is essential for safe perfusion even in the presence of a complete circle of Willis. Hypothermic circulatory arrest can be initiated when required. Frozen or conventional elephant trunk insertion or debranching for subsequent stenting is optional. When ligation of the left subclavian artery is required, an extra-anatomical bypass between the left subclavian and the common carotid artery with a Dacron graft may be necessary. If adequate collateral circulation supplies the left upper limb, such bypass may be futile. Finally, the aim remains to perform the distal anastomosis within zone 2 according to previously defined anatomical landmarks [42]. Needless to say, the assessment of the anatomy of the carotid arteries and the circle of Willis remains of paramount importance.

We did not observe paraplegia or spinal cord ischaemia.

The three patients who did not survive deserve some further discussion. Patient 8 initially recovered after a very stormy postoperative course, but she developed an extensive haemorrhagic stroke on the day before her planned discharge from which she never recovered. Again, patient 3 was ready for home discharge when he sustained a cardiac arrest following the rupture of his abdominal aorta. Patient 9 remained critical in intensive care due to a severe systemic inflammatory reaction leading to a fatal outcome on day 2 postoperatively. It is likely that the underlying vascular fragility in patient 8 (giant cell arteritis) and in patient 3 (Loeys-Dietz syndrome) may have played a role. The argument here is the care we can deliver to these patients as a multi-skilled team. If we set our frame of mind to the point that we must save everybody, then we are bound to fail. If we set ourselves in a way that we want to give the best possible care as a multi-skilled team, then we can save many and are likely to succeed. It is very convenient to talk about the successful outcomes, but it is what may have gone wrong that may lead to further improvements with achievement of the desired outcome later. Although we have used the technique for the past ten years, we have selected this case series mainly for the purposes of describing the technique and its feasibility. Therefore, the 30-day mortality in this context has to be acknowledged within certain limits.

The aim of this work was to discuss a different approach to complex patients as an additional option to the surgical armamentarium based on a multi-skilled team with a view to deliver the best possible care and achieve a successful outcome. The next stage would be a more complete review of all patients and possibly a comparison in a prospective manner with other approaches.

## 5. Conclusions

The approach described here may have a role for complex aortic arch procedures requiring prolonged cerebral protection. Although this alternative approach is well established in our current practice with a good technical success rate, our early experience should be subject to scrutiny and strict follow up in a larger registry (or in a randomised trial in comparison to the current standard).

## Figures and Tables

**Table 1:** Clinical and operative data: LVEF (left ventricular ejection fraction); LVIDd (left ventricular internal diastolic diameter); PASP (pulmonary artery systolic pressure); TAPSE (tricuspid annular plane systolic excursion); HTN (hypertension); DM (diabetes mellitus); CVA (cerebro-vascular accident); LAD (left anterior descending coronary artery); CPB (cardio-pulmonary bypass); X-Clamp (aortic cross-clamp); HCA (hypothermic circulatory arrest); AVR (aortic valve replacement); CoA (aortic coarctation).

Age	Diagnosis	Comorbidities	Preoperative Data	Operation	Perfusion Data	Outcome
42 years	Expanding pseudo-aneurysm of the ascending aorta with severe sternal adhesions and fistula formation. Previous AVR with aortic homograft for bicuspid aortic valve disease. Redo AVR with aortic root remodelling for homograft degeneration.	Previous CVA	LVEF 57%	Replacement of the ascending aorta with Dacron graft and partial resection of the sternum.	CPB 267 min X-Clamp 125 min T 25 °C	Alive at follow up.
60 years	Acute Type B aortic dissection with retrograde progression and involvement of the superior mesenteric artery.	HTN Chronic renal impairment on dialysis Sickle Cell anaemia	LVEF 45%; LVIDd 5.5 cm; PASP 34 mm Hg; TAPSE 2.5 cm; E/A ratio 2.3	Aortic arch replacement with frozen elephant trunk using Thoraflex device.	CPB 238 min X-Clamp 122 min HCA 73 min T 24 °C	Alive at follow up. TEVAR after 12 months for expanding false lumen in the descending thoracic aorta.
12 years	Type B aortic dissection with expanding false lumen in previous AVR with bioprosthesis and ascending aorta and arch replacement with Lupiae Dacron graft (11 years old). Previous valve sparing aortic root replacement in bicuspid aortic valve disease complicated by myocardial ischaemia requiring LAD patching (9 years old). Previous PDA device closure (1 year old).	Loeys-Dietz syndrome Renal impairment	LVEF 51%; LVIDd 4.1 cm	Replacement of distal aortic arch and proximal descending thoracic aorta with 22 mm Dacron graft. Readmission after four months for replacement of distal thoracic and abdominal aorta due to laminar thrombus in the abdominal aorta.	CPB 166 min T 32 °C	Deceased after 53 days postoperatively due to cardiac arrest following abdominal aortic rupture.
20 years	Aneurysm of the proximal descending thoracic aorta in previous ascending aorta and arch replacement with frozen elephant trunk.	Marfan’s syndrome HTN	LVEF 49%; LVIDd 6.1 cm; PASP 20 mmHg; TAPSE 1.35 cm; E/A ratio 1.2	Replacement of descending thoracic aorta with 34 mm Dacron graft; debranching of left subclavian artery with by-pass between left subclavian artery and left common carotid artery using 8 mm Dacron graft.	CPB 76 min X-Clamp 66 min T 34 °C	Alive at follow up.
43 years	Severe bicuspid aortic valve stenosis and narrowing of Dacron graft between ascending and descending thoracic aorta in previous redo CoA repair (aged 18). Previous end-to-end repair of CoA (aged 3) followed by resection of sub-aortic stenosis (aged 7).	Previous stroke; epilepsy	LVEF 69%; LVIDd 4.2 cm; PASP 29 mmHg; TAPSE 2.3 cm	Aortic valve replacement with 23 mm mechanical prosthesis and additional interposition Dacron graft between ascending and descending thoracic aorta.	CPB 167 min X-Clamp 90 min HCA 13 min T 16 °C	Alive at follow up.
85 years	Aneurysm of the ascending aorta and distal aortic arch (8 cm) in previous acute type B aortic dissection treated conservatively.	HTN	LVEF 65%; LVIDd 5.4 cm; PASP 25 mmHg; TAPSE 2.1 cm; E/A ratio 0.71	Replacement of the ascending aorta and debranching of the aortic arch to create a landing zone for a subsequent TEVAR procedure.	CPB 104 min X-Clamp 44 min T 34 °C	Alive at follow up. TEVAR after 2 months.
44 years	Severe aneurysmatic dilatation of the distal aortic arch and proximal descending thoracic aorta with compression of the left branch of the pulmonary artery.	HTN; DM Behcet’s disease	LVEF 62%; LVIDd 5.3 cm; PASP 36 mmHg; TAPSE 3 cm; E/A ratio 1.2	Replacement of the aortic arch with frozen elephant trunk using Thoraflex device.	CPB 265 min X-Clamp 70 min HCA 48 min T 18 °C	Alive at follow up.
51 years	Infected Dacron graft in the ascending aortic position with contained rupture and pseudo-aneurysm formation adherent to the sternum. Previous David’s procedure for ascending aortic aneurysm. Progression of the disease to the aortic arch requiring frozen elephant trunk with Thoraflex device 3 years later followed by TEVAR after 6 months.	HTN Renal impairment Giant Cell Arteritis	LVEF 55%; LVIDd 4.3 cm; TAPSE 1.3 cm; E/A ratio 1.2	Replacement of the infected Dacron graft in the ascending aortic position with an aortic homograft.	CPB 263 min X-Clamp 70 min HCA 49 min T 17 °C	Deceased after 26 days postoperatively due to severe haemorrhagic stroke.
75 years	Severe dilatation of the aortic arch (10 cm), ascending aorta (9 cm) and proximal descending thoracic aorta.	HTN; critical LAD disease	LVEF 45%; LVIDd 6.7 cm PASP 35 mmHg; TAPSE 2.2 cm	Replacement of ascending aorta and aortic arch with conventional elephant trunk using a 34 mm Siena Dacron graft and vein graft to LAD.	CPB 275 min X-Clamp 114 min T 17 °C	Deceased after 2 days postoperatively due to severe inflammatory reaction.
39 years	Severe aortic regurgitation with significant dilation of the aortic root (5.2 cm) and arch involvement. Repair of acute type A aortic dissection with interposition Dacron graft to the ascending aorta and resuspension of the aortic valve cusps 6 years earlier.	HTN	LVEF 66%; LVIDd 5.7 cm; PASP 10 mmHg; TAPSE 1.9 cm; E/A ratio 1	Modified Bentall procedure with composite mechanical graft and frozen elephant trunk with Thoraflex device.	CPB 276 min X-Clamp 127 min HCA 41 min T 18 °C	Alive at follow up
51 years	Severe aneurysmatic dilatation of the distal aortic arch.		LVEF 67%; LVIDd 5.4 cm; PASP 32 mmHg; TAPSE 3.3 cm; E/A ratio 1.4	Replacement of ascending aorta, aortic arch and proximal descending thoracic aorta with Dacron graft using conventional elephant trunk.	CPB 437 min X-Clamp 21 min T 28 °C	Alive at follow up.
56 years	Expanding diameter and false lumen in previous acute type B aortic dissection treated conservatively.	HTN	LVEF 74%; LVIDd 5.6 cm; PASP 16 mmHg; TAPSE 2.6 cm; E/A ratio 1.1	Aortic arch replacement with frozen elephant trunk using Evita device.	CPB 228 min X-Clamp 55 min HCA 34 min T 24 °C	Alive at follow up.

## Data Availability

No additional data or links to other sources are available.
